# Calibration methods to fit parameters within complex biological models

**DOI:** 10.3389/fams.2023.1256443

**Published:** 2023-10-18

**Authors:** Pariksheet Nanda, Denise E. Kirschner

**Affiliations:** Department of Microbiology and Immunology, University of Michigan Medical School, Ann Arbor, MI, United States

**Keywords:** multi-scale modeling, hybrid models, non-linear, stochastic, datasets, spatial, dynamical, Bayesian

## Abstract

Mathematical and computational models of biological systems are increasingly complex, typically comprised of hybrid multi-scale methods such as ordinary differential equations, partial differential equations, agent-based and rule-based models, etc. These mechanistic models concurrently simulate detail at resolutions of whole host, multi-organ, organ, tissue, cellular, molecular, and genomic dynamics. Lacking analytical and numerical methods, solving complex biological models requires iterative parameter sampling-based approaches to establish appropriate ranges of model parameters that capture corresponding experimental datasets. However, these models typically comprise large numbers of parameters and therefore large degrees of freedom. Thus, fitting these models to multiple experimental datasets over time and space presents significant challenges. In this work we undertake the task of reviewing, testing, and advancing calibration practices across models and dataset types to compare methodologies for model calibration. Evaluating the process of calibrating models includes weighing strengths and applicability of each approach as well as standardizing calibration methods. Our work compares the performance of our model agnostic Calibration Protocol (CaliPro) with approximate Bayesian computing (ABC) to highlight strengths, weaknesses, synergies, and differences among these methods. We also present next-generation updates to CaliPro. We explore several model implementations and suggest a decision tree for selecting calibration approaches to match dataset types and modeling constraints.

## 1. Introduction

**FIGURE 1:**
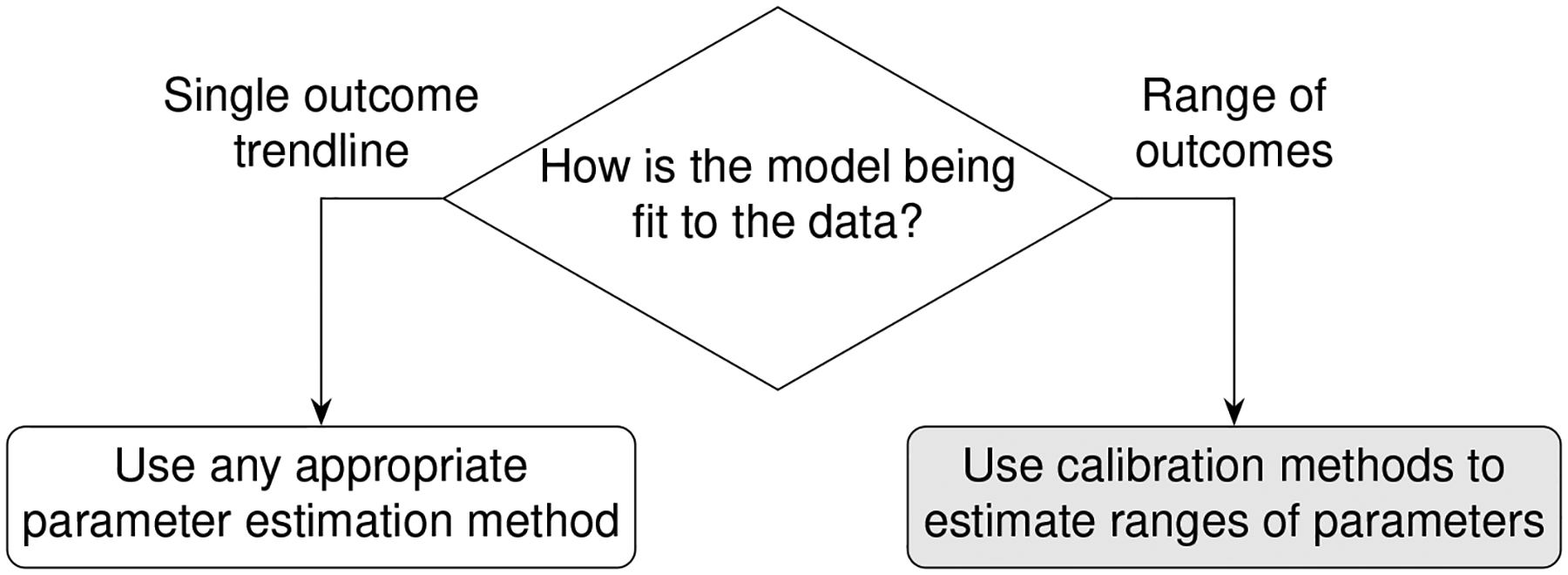
Calibration differs from traditional parameter estimation goals. The outcome of calibration is to fit to a range of experimental data. The goal is to adjust the set of parameter range boundaries to loosely limit model simulations to the biological boundaries of the reference experimental datasets.

Building computational and mathematical models to simulate complex non-linear biological processes requires many key steps in defining both the model as well as identifying ranges of values for many corresponding parameters. Complex models often require concurrent estimation of dozens of parameters using reference datasets derived from biological experiments. However, the step of identifying relevant ranges of parameter values in a complex model complicates the step of parameter estimation because traditional methods that find parameter point estimates are not useful and instead parameter estimation methods must identify ranges of biologically plausible parameter values. For example, models built to study infectious diseases would require parameter ranges wide enough to produce biological variation that would span healthy and disease outcomes. This atypically broad objective function of finding multiple solutions is sometimes referred to as suboptimal non-linear filtering [1]. In this paper, identifying acceptable ranges of model parameters is called calibration which is contrasted against traditional parameter estimation in Figure 1.

The choice of calibration method depends on the reference experimental datasets as well as the model type being calibrated. Calibrating to reference experimental datasets such as dynamical and/or spatial experimental datasets are discussed in the next section. The remaining sections cover the calibration of different model types. Models that have tractable likelihood functions and therefore are non-complex, do not require the methods discussed in this paper. Models of ordinary differential equations (ODEs) that fall under this non-complex scenario are briefly discussed. Complex models contain many structurally unidentifiable parameters requiring particular attention to calibration [2]. For the remaining complex models such as complex ODEs, partial differential equations (PDEs), agent-based models (ABMs), hybrid models, etc., we provide a decision tree to suggest which calibration method is most appropriate by examining both model type as well as characteristics of corresponding datasets. We explore three calibration methods, namely, calibration protocol (CaliPro), approximate Bayesian computing (ABC), and stochastic approximation. To aid discussion of this wide range of methods, we provide descriptions of phases and keywords for quick reference (see Table 1).

### 1.1. Characteristics of reference experimental datasets

A defining feature of complex biological systems is their incomplete, partially observable, and unobservable datasets. The uncertainty of incomplete and partially observable experimental results favors fitting model simulations to the boundaries of such datasets more often than considering modes within their distribution ranges as significant. Therefore, the limitation of these partial datasets justifies calibrating to ranges of data rather than to individual data points. On the other hand, unobservable data require modelers to assign parameters representing biological processes that may not be experimentally validated, but whose parameter ranges need to be calibrated alongside other experimental datasets. The number of non-experimentally bound parameters typically exceed the parameters that can be directly bound to available datasets, creating many degrees of freedom in the calibration process.

Several types of reference experimental datasets such as numerical, categorical, temporal, spatial, and synthetic datasets may be used to calibrate complex models. Numerical datasets can either be continuous or discrete and are well supported by inference methods even when missing data [6–8]. Calibrating a dynamical model to temporal datasets typically requires several comparisons along simulated trajectories to the temporal datasets. Calibrating a model to spatial datasets requires finding appropriate numerical or categorical summary statistics that may include image pre-processing steps to identify and match features of interest [9–11].

### 1.2. Calibrating non-complex models with numerical likelihood evaluation

Non-complex models, such as biological models that are comprised of systems of ordinary differential equations (ODEs), it is much easier to identify ranges for parameter values from corresponding datasets. Likelihood functions describe the joint probability of observed datasets and the chosen model. Thus, evaluating the likelihood function directly links model parameters with experimental data and guides the calibration process to directly identify parameter ranges. Likelihood calculation is central to probabilistic modeling [12]. Although likelihood-based parameter estimation methods are typically used when analytical solutions are not available, likelihoods can be found for such models to calibrate their parameters. For example, ODEs can produce exact probability density functions using the method of characteristics, which are used to calculate their likelihood [13]. However, calculating likelihood becomes unobtainable for complex models therefore requiring a different approach.

### 1.3. Background concepts to calibrate complex models

**FIGURE 2:**
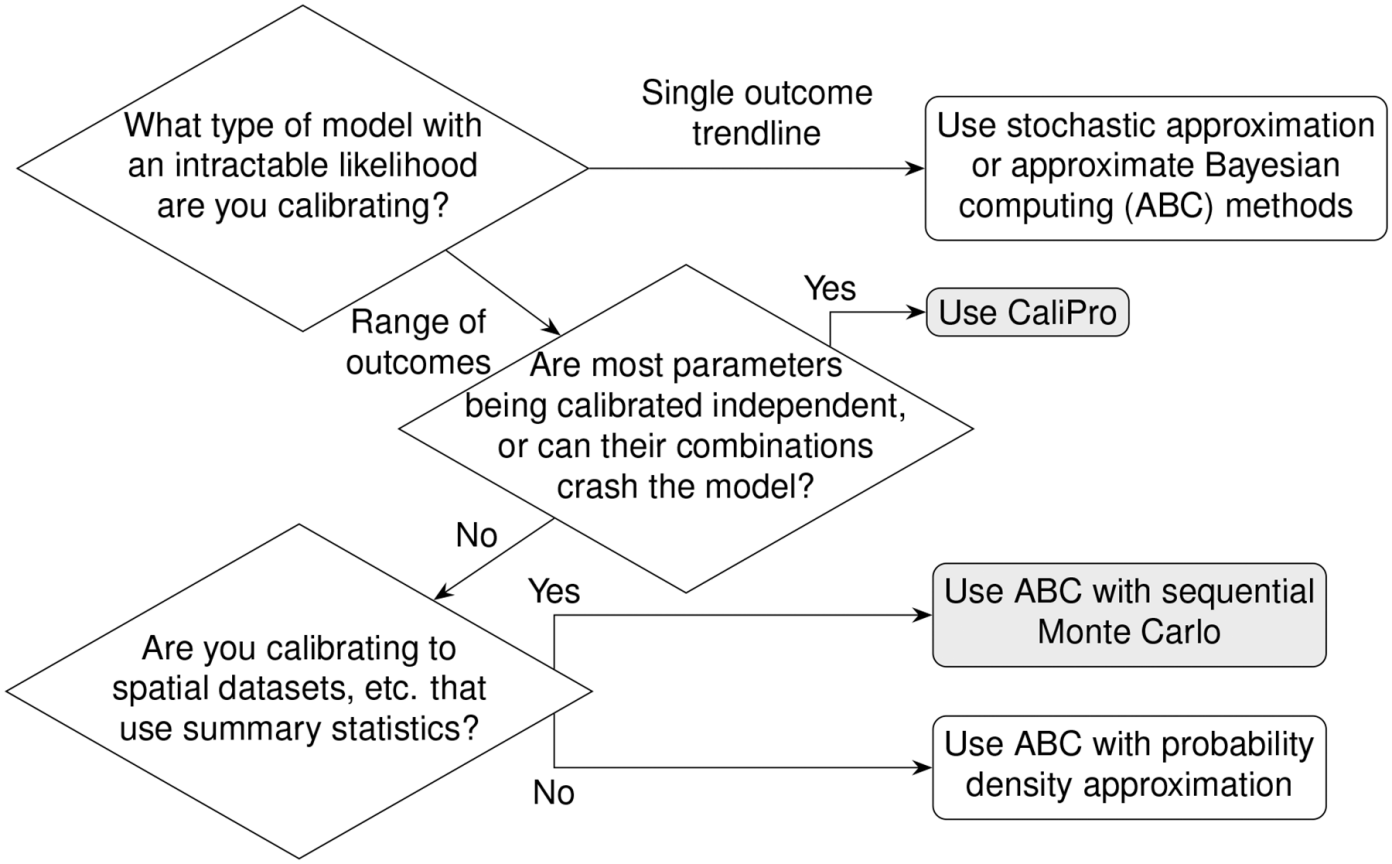
Decision tree for calibration. Calibrating models to datasets heavily depend upon model behaviors, dependencies between calibration parameters, and type(s) of data available for calibration. Calibration without dimension reduction or distance evaluations is possible with CaliPro and with the probability density approximation variant of approximate Bayesian computing. The methods are detailed as follows: stochastic approximation is detailed in Section 1.3.1, CaliPro is detailed in Section 1.4.1, ABC with sequential Monte Carlo is detailed in Section 1.4.2, and ABC with probability density approximation is detailed in Section 1.4.2.1.

Above we described the case for models that can obtain parameters using a likelihood function determined from direct fits to data. In the next section we will review two methods that estimate parameter ranges for complex biological systems where likelihood functions are not obtainable. We describe two published methods, the Calibration Protocol (CaliPro) [14] and approximate Bayesian computing (ABC) [15–18]. In this work, our goal is to review these model calibration methods and to compare and contrast them. We first set up background information that is applicable to both approaches and then provide more detail for each approach via examples. We provide a decision tree to help guide which approach to use (Figure 2).

#### 1.3.1. Inapplicability of stochastic approximation

Although stochastic approximation methods can also be used in contexts when the likelihood function is unavailable, this method does not directly serve our purpose of calibration. Stochastic approximation either uses a variant of finite differences to construct a stochastic gradient or stochastic perturbations that are gradient-free [19, 20]. However, both variants attempt to converge to an approximate maximum likelihood and then find the variance around the converged result using bootstrap. This is not the same as the intended calibration goal of preserving broad parameter sampling around parameter space containing multiple solutions that fit the experimental data.

#### 1.3.2. Sampling outcome and parameter spaces

**FIGURE 3:**
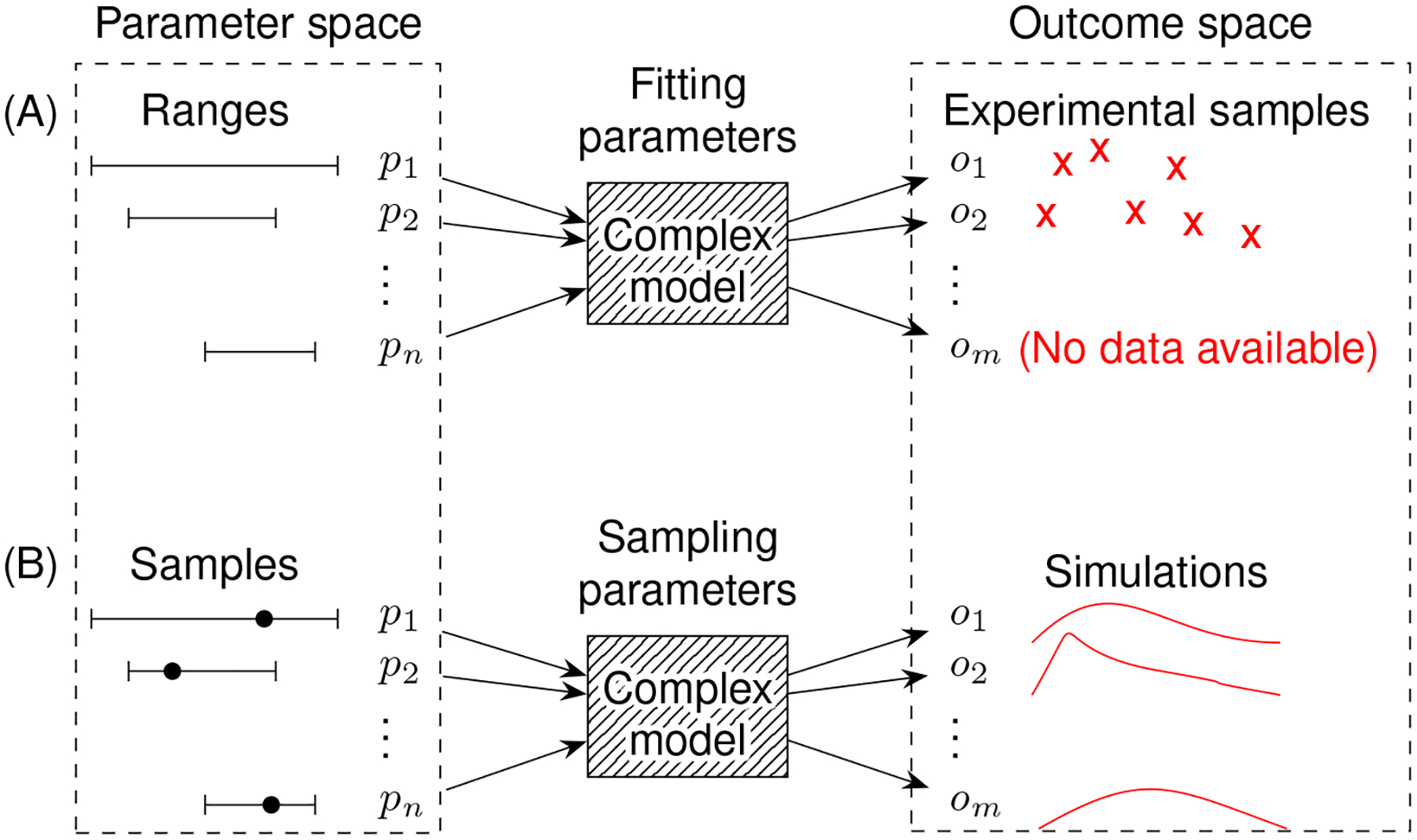
Complex model simulations connect samples in parameter space to samples in outcome space. In addition to parameter space, there also exists high dimensional outcome space, connected by simulations from a complex model. A model with parameters p_1_…p_n_ produces outputs o_1_…o_m_. **(A)** Experimental samples help set putative parameter ranges. However, not all model outputs may be practical to sample experimentally. **(B)** Simulations generated by the model from sampled parameter ranges try to match the experimental samples.

Sampling experimental datasets can be thought of as sampling multidimensional outcome space (Figure 3). Datasets may not be available for some outcomes and thus increase the burden of parameter sampling. The challenges of limited datasets and high-dimensional parameter and outcome spaces motivate the need to use careful parameter sampling schemes.

#### 1.3.3. Parameter sampling schemes

Complex models with their many parameters can be thought of as forming a hypercube of high-dimensional parameter space. Increasing the number of model parameters or dimensions exponentially increases the combinatorial complexity of visiting parameter space on an evenly spaced grid of a discretized parameter hypercube.

**FIGURE 4:**
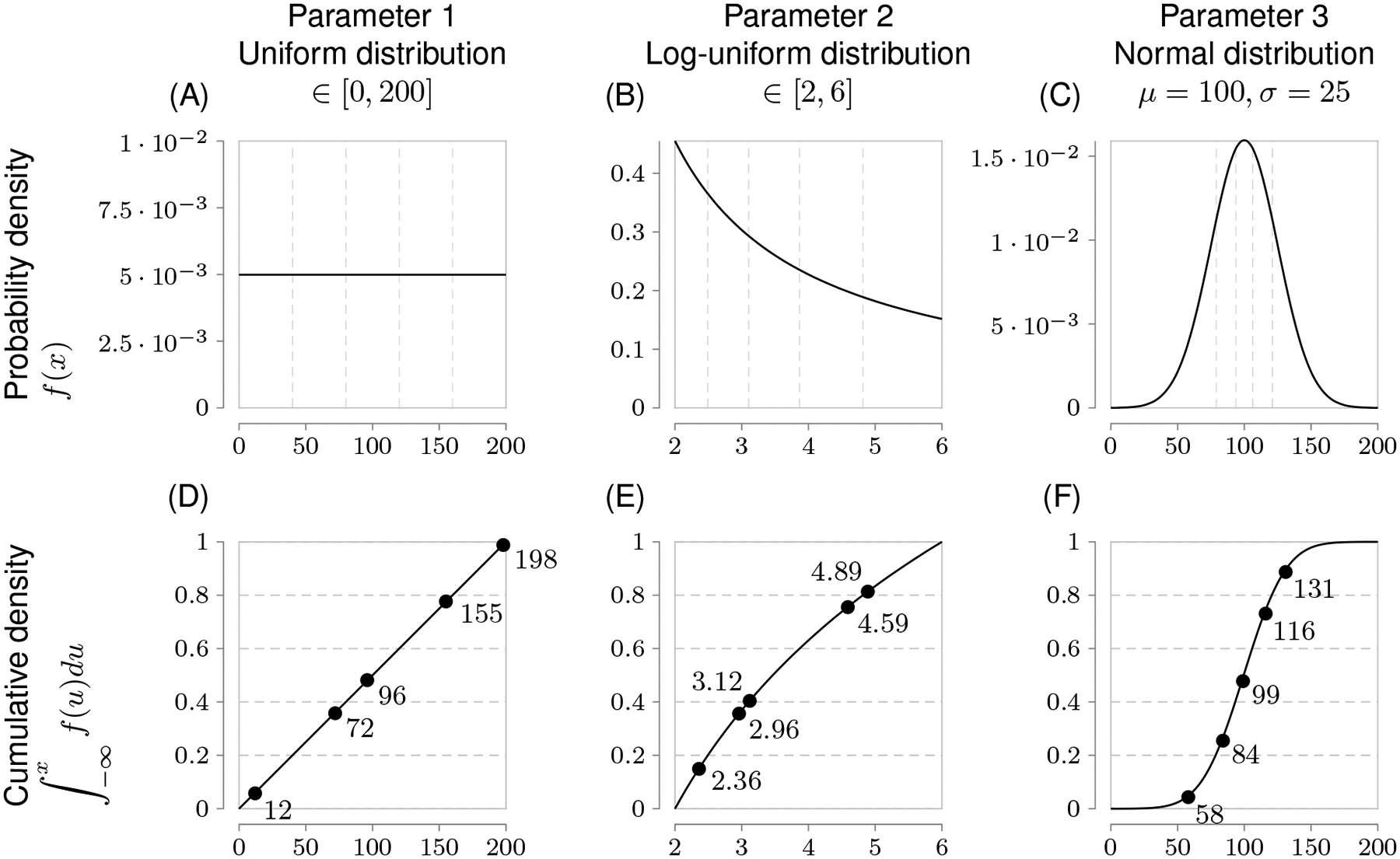
Globally sampling parameters evenly using the cumulative probability density transformation. The probability density function (PDF) is a line under which the area is 1. Parameters can have different types of PDFs such as uniform, log-uniform and normal distributions shown in **(A–C). (A)** Shows a simple uniform distribution. The log-normal distribution helps sample parameters with wide ranges and in this example in **(B)** the x-axis represents the exponent, namely Parameter 2 varies from 10^2^ to 10^6^. The shape of the normal distribution is set by the *μ* (mean) an *σ* (standard deviation) values. However, non-uniform PDFs **(B, C)** have their densities concentrated at high probability regions as shown by the uneven vertical grid spacing in **(B, C)** that mark 5 regions each of 20% probability. To evenly sample from these non-uniform distributions, it is first necessary to generate the cumulative density function (CDF) which evens out the probability space in the y-axis as shown by the corresponding horizontal grid lines in **(D–F)** corresponding to the original 5 regions each of 20% probability. Random samples drawn from each of the 5 probability intervals [0.0, 0.2), [0.2, 0.4),[0.4, 0.6), [0.6, 0.8), and [0.8, 1.0] on the y-axis of the CDF are represented by solid circles and their x-axis parameter values are labeled next to the solid circles. Randomly sampled parameter values have been rounded for easier reading.

However, an evenly and finely spaced parameter grid required to sample these regions adequately in parameter space is not necessarily linear. Each parameter has an associated probability value for a particular parameter value. Only uniform probability distributions are linear for a given range because any value of such a parameter within the bounds of the uniform distribution has the same probability. On the other hand, parameters with non-uniform probability distributions require generating samples in accordance with their cumulative probability density (Figure 4). This allows measurements to better capture characteristic skewness, etc., to adjust for inferring the true parameter distribution.

Another consequence of high-dimensional parameter space is that it cannot be exhaustively sampled. Thus, sampling methods need to strategically stratify the space and choose parameter values for a particular calibration method. The two main strategies are global and local sampling. Global sampling schemes such as Latin hypercube sampling (LHS) (Table 2), Sobol sampling, and random sampling—also called Monte Carlo sampling—provide a means of broadly exploring parameter space, studied extensively in Renardy et al. [22]. On the other hand, local sampling schemes depend on previously sampled values to suggest future values. We review such local sampling schemes in more detail in the section on Approximate Bayesian Computing.

#### 1.3.4. Parameter sampling with pseudo-likelihood evaluation

Complex models lack both closed-form analytical solutions and numerical approximations of likelihood, which restricts parameter range estimation methods to iterative parameter sampling. Between iterations many parameters need to be varied and the model outcomes need to be continuously re-evaluated for goodness of fit to available experimental datasets.

**FIGURE 5:**
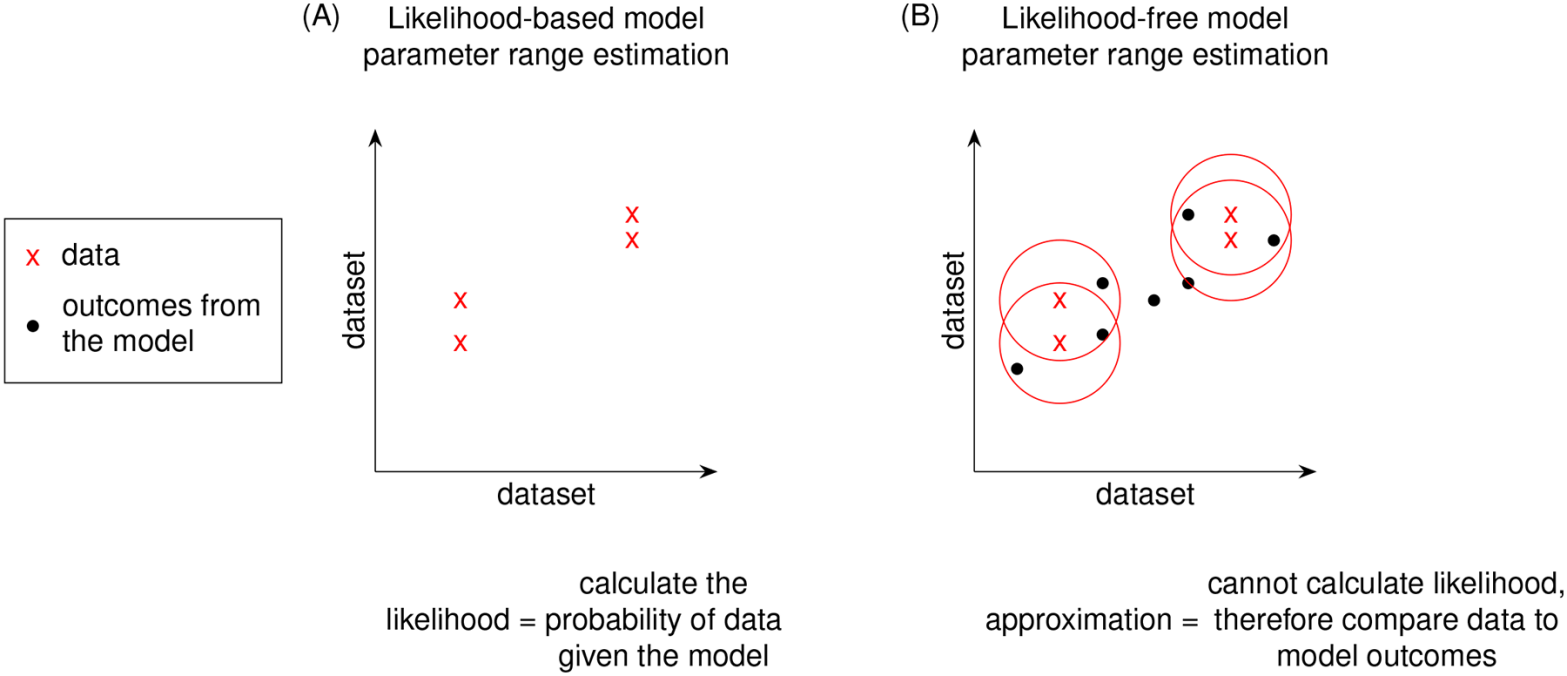
Using a distance function to replace likelihood. We replace likelihood functions with a distance function between data and model outcomes to avoid numerical evaluation. For models where it is not feasible to evaluate their likelihood function, model-generated outcomes can instead be compared to experimental data to approximate the likelihood function by penalizing sampled-parameter draws that predict model outcomes farther away from experimental data. The graphs shown in **(A, B)** are a simple 2 dataset model. Models with greater dataset dimensions can be similarly visualized in two dimensions using a t-statistic stochastic neighbor embedding (t-SNE) plot [23]. The red circles in **(B)** represent a distance function for comparing the data to model outcomes.

**FIGURE 6:**
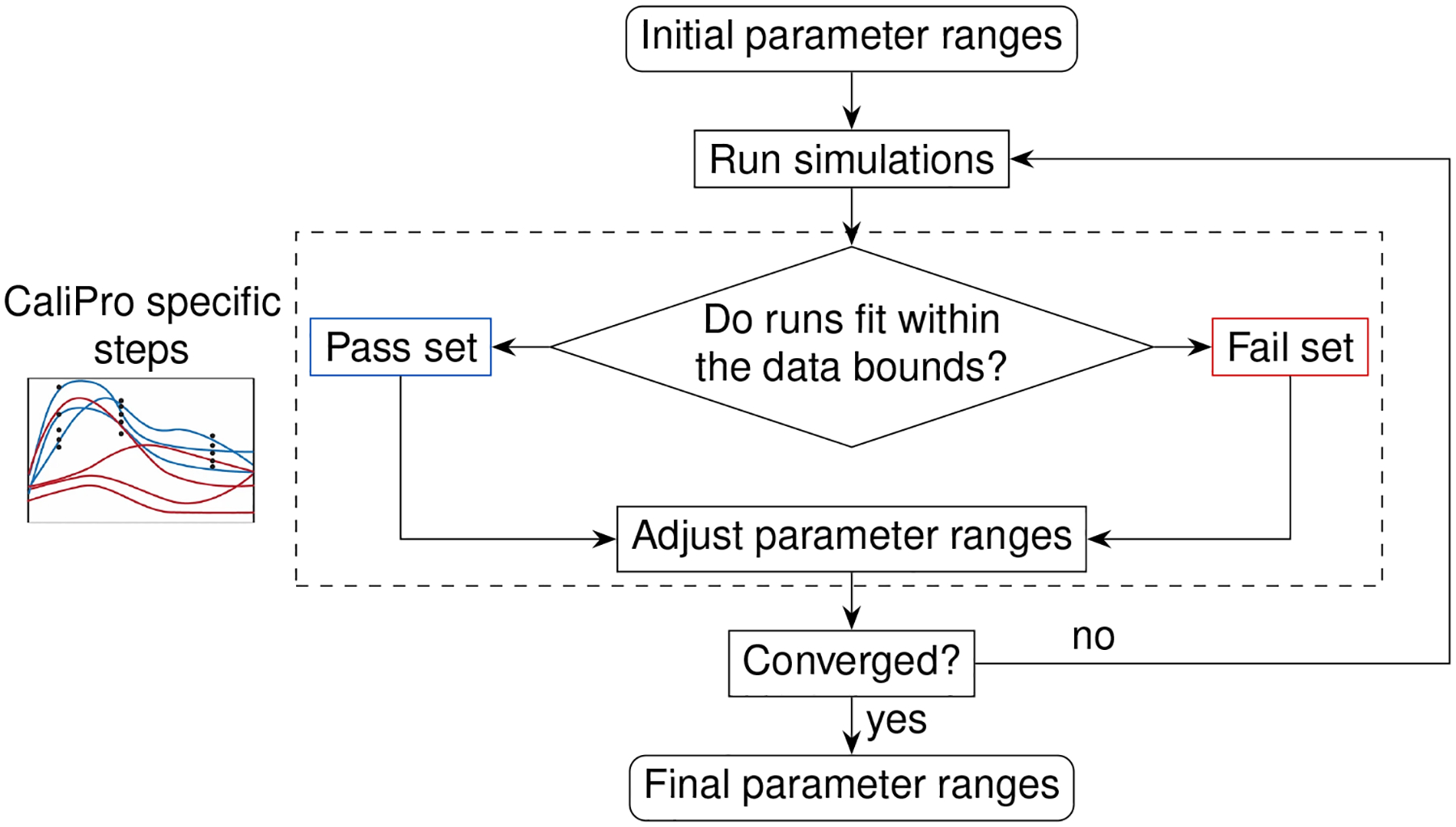
Evaluation loop of our Calibration Protocol (CaliPro). The schematic shows the evaluation steps of CaliPro, which consists of a pass-fail run classification that we use for adjusting parameter ranges. Unlike the Bayesian method which generates parameter distributions, CaliPro adjusts parameter ranges using either alternative density subtraction (ADS) using both the pass and fail parameter densities, or the highest density region (HDR) using only the pass set if the parameter started with a narrow range and low variation. Left box: simulation trajectories (curves) overlays with data points (black dots) colored as blue (pass set) and red (fail set).

For non-complex models, goodness of fit to experimental data would involve likelihood functions as described above (and in Table 1). However, complex models do not have tractable likelihood functions and therefore require alternative methods of comparing to experimental datasets. Instead of evaluating likelihood functions, methods for fitting complex models rely on comparisons to experimental data (Figures 5, 6). Such pseudo-likelihood evaluations are therefore used for fitting complex model parameters.

### 1.4. Methods to calibrate complex models

Now that we have narrowed the scope of calibration to models requiring pseudo-likelihood functions, as well as observed the need for iterative sampling to fully explore data fits for parameter space, we will detail two broad classes of methods we use to accomplish this task: CaliPro and ABC. CaliPro is introduced first because it is the more intuitive of the two methods and derives from published work from our own group.

#### 1.4.1. Calibration protocol

We recently formalized a calibration protocol (CaliPro) method to calibrate complex biological models while also remaining agnostic to model type [14]. The goal of CaliPro is to adjust parameter boundaries to capture large and disparate datasets using a minimal number of iterations to converge to an acceptable fit. CaliPro classifies simulations into *pass* and *fail* sets (Figure 6). We use both pass and fail classifications of model simulations to adjust parameter boundaries using CaliPro’s alternative density subtraction (ADS) method. Alternatively, for parameters with smaller ranges and less variance, the highest density region (HDR) parameter adjustment method provides faster convergence because HDR adjusts parameter ranges to regions of higher probability density whereas ADS first subtracts the probability density of fail sets to output smaller changes. Finally, we use Boolean function thresholds to define pass rates. In practice, >75–90% of simulations passing is sufficient to end calibration, as over constraining the convergence function risks overfitting parameters.

Using CaliPro in practice requires attention to several details. To establish model-specific pass-fail constraints of model simulations, we require *a priori* knowledge of the biological system and thus this step is based on user discretion. We previously discussed several examples of establishing such pass-fail constraints [14]. Secondly, when we first use CaliPro or after making significant changes to the model, the pass rate may be very low and may not improve after several iterations. This is because the low pass rate is too uninformative for the parameter range adjustment method to propose useful new parameter ranges. In such cases, the more stringent among the constraints employed should be disabled after measuring the pass set from each individual constraint and then those stringent constraints can be reapplied later after achieving higher pass rates. Thirdly, to calibrate large numbers of unknown parameters, one can reduce the number of fail set outcomes by paying attention to starting values of the most sensitive parameters. The most sensitive parameters that affect the model can be determined using partial rank correlations (PRC) [21]. Lastly, for stochastic models that have variable outcomes even with fixed parameters, the number of pass sets can plateau in an undesirable part of parameter space therefore requiring multiple starting seeds for at least some simulations to converge.

A larger framework to which CaliPro belongs for complex model calibration is approximate Bayesian computing (ABC) because of the many similarities between the independently developed techniques. Approximate Bayesian computing was mentioned in Figure 2 of the CaliPro method paper [14], but the techniques were not directly compared. This paper helps address that gap.

#### 1.4.2. Approximate Bayesian computing

Approximate Bayesian computing (ABC) works around the difficulty of numerically evaluating model likelihood functions by simulating model outcomes and then often applying a distance function or a kernel density estimator to compare to reference datasets (Figure 5).

ABC requires a parameter sampling strategy to generate distributions of parameters of interest. Nearly all sampling strategies used in practice for ABC are techniques that sample around previously sampled locations using Markov processes and weights that are iteratively updated to guide subsequent sampling [18]. Sequential Monte Carlo (SMC) sampling uses hidden states to affect a slowly changing distribution to efficiently reach the true parameter distributions [18, 24]. Unlike most other Monte Carlo sampling schemes used in Bayesian inference where chains primarily measure the r-hat quality of sampled parameters, SMC chains accelerate exploration of parameter space and is therefore the sampling technique frequently used for ABC calibration.

##### 1.4.2.1. Summary statistics and their sufficiency

Summarizing model outcomes or experimental datasets is necessary in these pseudo-likelihood parameter sampling situations when the model output does not exactly match the type of data. Applying summaries to model outcomes or datasets allow them to be numerically compared. An example of a model summary statistic would be the total diameter of a tumor calculated from the corresponding model simulated spatial components.

For some applications, the general case of summarizing many outcomes of non-linear complex models is prone to inefficient inference or even non-convergence to the true parameter distribution when the summary statistics are not sufficient [25] as described in Table 1. The error introduced by not having sufficient summary statistics is not measurable because the likelihood function is not available [26]. Sufficient statistics are the property of summary statistics containing as much information as the parameter samples (see Table 1 for definitions). As mentioned, truly knowing whether a summary statistic is sufficient also requires a likelihood function, therefore this validation is impractical for a complex model [26]. If a summary statistic is not sufficient, convergence to the true parameter distribution is not guaranteed. As a workaround, one can use probability density approximation (PDA) to avoid using summary statistics [25].

Due to this risk of insufficiency when using summary statistics, it is preferable to avoid using summary statistics or to limit their use to cases that require it, such as calibrating to spatial datasets, where simulations, for example, of cell type ratios or intercellular proximities must be collectively expressed as summaries rather than raw counts. Summary statistics are used in the first place to reduce the model outcome dimensionality to compare with experimental datasets. Therefore, given the importance of sufficiency and difficulty of knowing the quality of summary statistics, it is useful to have alternative calibration methods that do not require using sufficient statistics such as our method, CaliPro, and also ABC-PDA [25].

In addition to using summary statistics, another strategy to improve convergence is to use a Markov process (described in Table 1). This improves fitting parameter ranges by smoothing the changing parameter distribution from its initial samples to the true distribution. Using a Markov process makes it efficient to process many parameter samples [18] and is the basis of sequential Monte Carlo sampling and sequential importance sampling. Both algorithms are widely used and are known in different fields by different names such as bootstrap filtering, the condensation algorithm, particle filtering, interacting particle approximations, survival of the fittest, and recursive Bayesian filtering [1]. Particle filtering is often used to describe these sampling algorithms; therefore, we define a particle in this context. Simulation outcomes are thought of as particles consisting of intersecting probability distributions of lower dimensional model parameter summaries. Particles have associated weights and those weights are used to iteratively resample or move particles to better approximate the true parameter distributions [27]. Thus, calibration requires using sampling techniques that can scale to a large number of parameter samples of complex models using particle filtering and Markov smoothing techniques.

## 2. Method

Here we outline how we perform both the CaliPro and ABC calculations. In our previously published work, CaliPro was only used to tune uniform distributions; to compare CaliPro more directly to ABC, here we extend CaliPro’s uniform distribution boundaries to non-uniform distributions. We do this by fitting non-uniform distribution parameters using both the boundaries along with their globally sampled percentiles. To perform ABC, we used the PyMC package [28] with the Metropolis–Hastings kernel. We also tried using the pyABC package [29], but each calibration attempt ran out of memory even on large memory computer clusters. The conceptual Figures 1–6 were creating using LaTeX with the PGF-TikZ package [30, 31]. For the remaining figures, all example models with their associated data, commented code, and output files are archived on Zenodo [32].

### 2.1. Calibration protocol with Latin hypercube sampling

To improve useability and understanding of LHS and CaliPro, these general methods have been implemented in R using the lhs package [33]. The CaliPro pass–fail criteria are described in the results section for each of the models. No termination pass percentage was used and instead calibration was allowed to continue for a pre-determined number of iterations.

All parameter updates are done using the alternative density subtraction (ADS) algorithm. ADS outputs parameter boundaries are originally intended for uniform distributions. To extend ADS to other types of distributions, we use the LHS percentiles sampled along with the new distribution drawn boundaries to fit the parameter distributions between iterations (see Section 2.1.1).

#### 2.1.1. Fitting probability distributions using both percentiles and distribution draws

Probability distributions are conventionally fit using many distribution draws. However, both CaliPro’s HDR or ADS algorithms provide uniform distribution boundaries as outputs, which we then need to fit to non-uniform distributions. To meaningfully use the limited two distribution draws of the boundaries, we also need to know the percentiles to which those data points belong. This idea of using both the distribution draws and their percentiles is also useful for setting initial parameter distributions from biological journals and clinical trial datasets that are often reported in the form of 3 data points: the median and interquartile range, which together provide distribution draws for the 25, 50, and 75% quantiles. This was necessary for fitting distributions to the parameters of the immune-HIV-1/AIDS example model. Reporting experimental parameters using quantiles implies that distributions cannot be fit in the conventional way using maximum likelihood of a large collection of distribution draws. Instead, we use both the known distribution draws *x*, and their corresponding known percentiles, *p*, to fit the unknown distribution parameters, $\hat{\theta }$ , using optimization. We supply percentiles, *p*, with the estimated distribution parameters, $\hat{\theta }$ , to the inverse cumulative density function to obtain estimated distribution draws, $\hat{x}$, and then compare $\hat{x}$ against the known distribution draws, *x*, to minimize the prediction error while tuning $\hat{\theta }$. We detail the corresponding equations for obtaining this distribution fit below. We used the L-BFGS-B bounded optimization method [34] implemented by the *optim*() function of the stats R package [35] to fit the distribution parameters.


$$F\left(x,\theta \right)=\int_{-\infty }^{x}f\left(t,\theta \right)dt=p\;Q\left(p,\theta \right)=F^{-1}\left(p,\theta \right)=x\;\text{Minimize : }\sum \left\|{\hat{x}}_{i}-x_{i}\right\|\;\forall i\in 1..n\;\text{Subject to : }{\hat{x}}_{i}=Q\left(p_{i},\hat{\theta }\right)$$

where,

f≡ Distribution probability density function (PDF)

F≡ Distribution cumulative density function (CDF)

Q≡ Distribution percentile function or inverse CDF

$x_{i}\equiv$ Known distribution draws

$p_{i}\equiv$ Known percentiles corresponding to known distribution draws

$\hat{\theta }\equiv$ Estimated distribution parameters that are being optimized/fit

${\hat{x}}_{i}\equiv$ Estimated distribution draws from the known percentile and estimated parameters

n≡ Number of known distribution draws with corresponding known percentiles.

#### 2.1.2. T-statistic stochastic neighbor embedding plots

The t-SNE coordinates were calculated using the Rtsne package [36–38]. These plots help visualize higher-dimensional parameter space sampled by LHS.

### 2.2. Approximate Bayesian computing with sequential Monte Carlo sampling

We ran the ABC-SMC inference method for the example models using the PyMC package [28] version 5.3.0 in the python programming language. For the immune-HIV-1/AIDS model, we customized the solver and distance comparison as follows:

1. We replaced the default SMC multivariate-normal kernel to the metropolis–hastings kernel as a workaround for crashes from incomplete model simulations.
2. Instead of comparing simulations against multiple patient CD4+ cell count timeseries, we chose only a single patient timeseries to avoid use of summary statistics due to hard-to-diagnose errors from deferred evaluations of the pytensor symbolic expressions when attempting to run a summary statistics function.
3. The patient timeseries is known to be non-progressive HIV infection. Therefore, to minimize the fixed error from the distance function while the simulation reaches steady state, the first 5 years of the simulation are omitted, and the 5th year onward is compared against the 10 years of patient timeseries.

## 3. Results

Our goal is to apply both CaliPro and ABC approaches to calibrate two different examples and compare them: a non-complex and complex model. The following models of ordinary differential equations (ODEs), will be evaluated: the classic predator–prey model [39, 40], and a viral–host response model of HIV-1/AIDS infection [41]. While stochastic models including agent-based models are of particular interest for these calibration techniques, directly calibrating such large models is beyond the scope of this work and instead we discuss these models using examples already published.

Finally, we will compare calibration performances of CaliPro-LHS against ABC-SMC to show practical strengths and weaknesses of each. These approaches will guide modelers to explore parameter space of complex non-linear models to incomplete experimental datasets.

### 3.1. Ordinary differential equation models

#### 3.1.1. Lotka–Volterra

The two-equation predator–prey ODE model [39, 40] is one of the simplest systems to evaluate fitting against noisy simulated data:

$$\frac{dx}{dt}=+\alpha x-\beta xy\;\frac{dy}{dt}=-\gamma y+\delta xy$$

where,

x≡ prey populationg

y≡ predator population

α≡ prey growth rate = 1.0 [per year]

β≡ prey death rate = 0.1 [per year]

γ≡ predator death rate = 1.50 [per year]

δ≡ predator growth rate = 0.75 [pear year]

Initial values:

$x_{0}\equiv$ initial prey population = 10.0

$y_{0}\equiv$ initial predator population = 5.0

Priors:

α~Half-Normal(μ=1.0,σ=1.0) [per year]

β ~ Half−Normal(μ=1.0,σ=1.0) [per year] (detuned from μ=0.1 training data)

γ=1.50 [pear year] (fixed)

δ=0.75 [per year] (fixed)

The simulated data instead uses the parameter *β* = 0.1 and adds random noise drawn from a standard Normal distribution. The uncalibrated prey death rate parameter *β* = 1.0 causes the prey population to crash early on and therefore the predator population to also crash; they only recover values close to their original population levels starting from year 5 onward. The uncalibrated population trajectories are shown by sampling from the prior distribution. We show results of varying and calibrating the *α* and *β* parameters to a noisy dataset using while keeping parameters *γ* and *δ* fixed.

**FIGURE 7:**
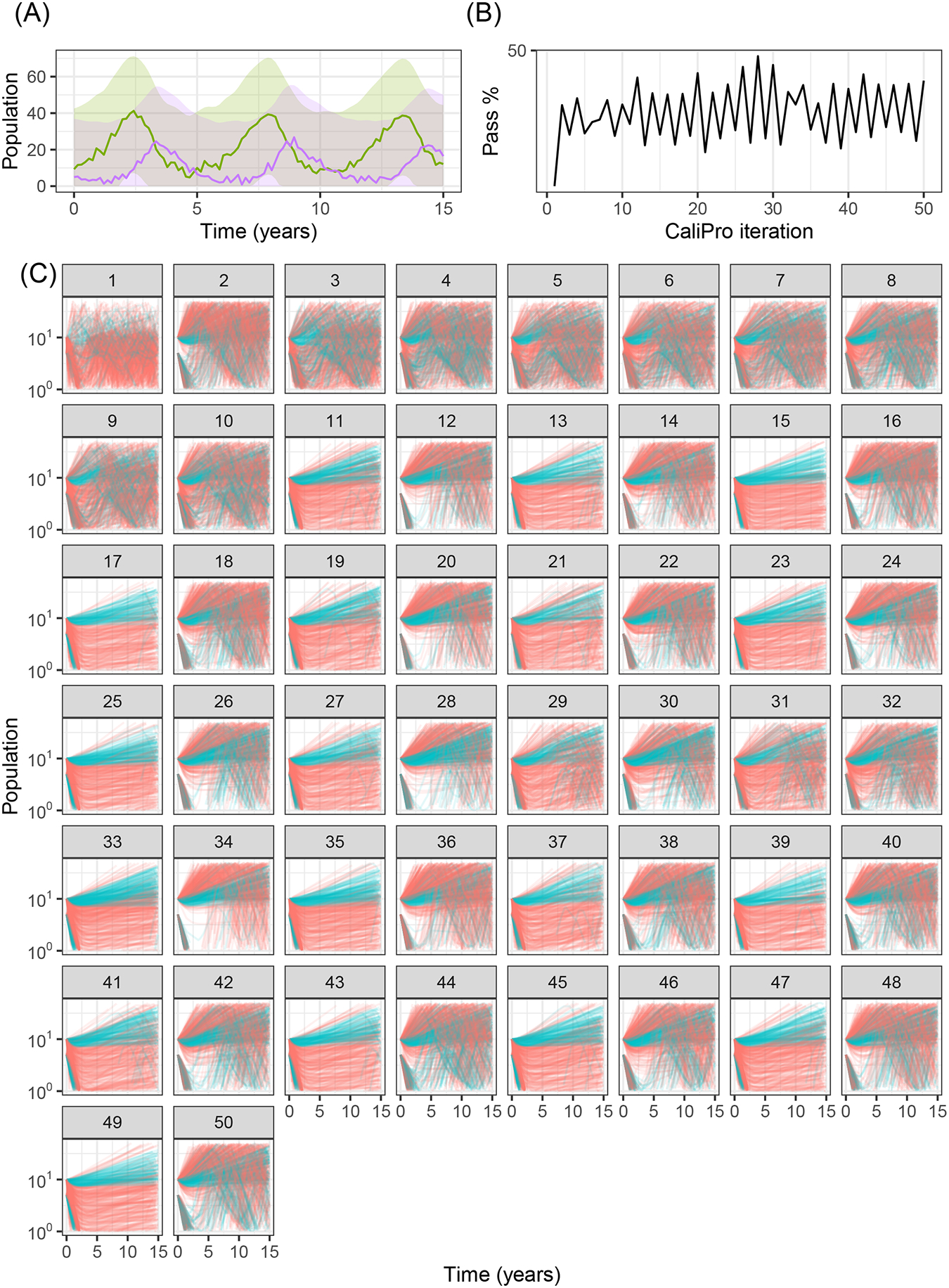
Calibration trajectories of Lotka–Volterra model using CaliPro. **(A)** We obtain CaliPro pass criteria shown in translucent shaded region by kernel smoothing to denoise the data and then relax the fit by 8 times the largest residual. Purple curves indicate predator population and green curves indicate prey population. **(B)** Pass percentages of simulation replicates start at 4% and reach a maximum of 51% by iteration 28. **(C)** We consider simulations to pass if all points of the trajectory are the CaliPro boundaries of **(A)**. Trajectories of simulations at later iterations show oscillation between the two varied parameters, because the parameters are dependent whereas LHS global sampling assumes parameters are independent. Calibrating this model with dependent parameters with CaliPro-LHS therefore requires one of the parameters to be fixed. The passing simulations are colored blue and failing simulations are colored red.

**FIGURE 8:**
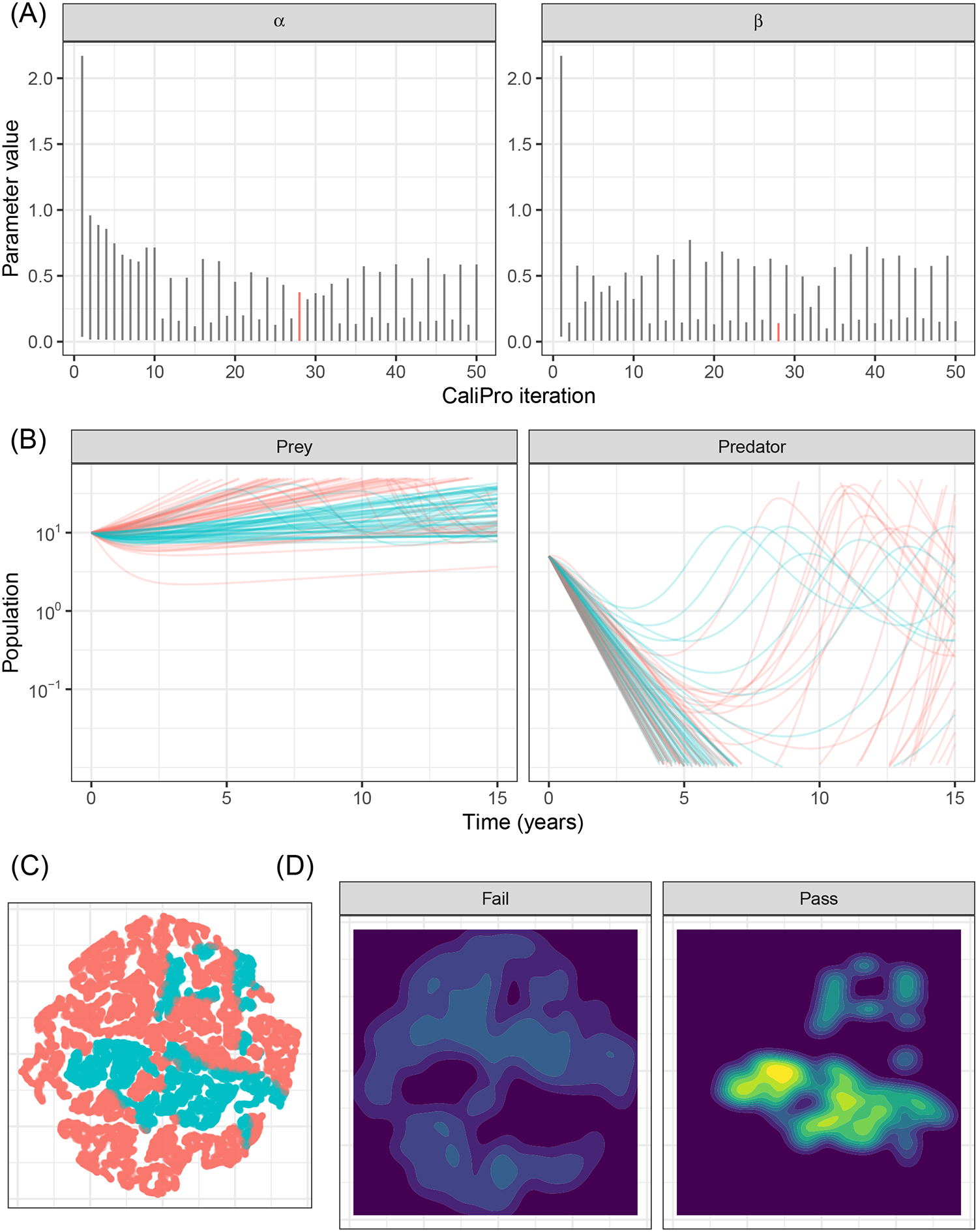
Calibration parameters of Lotka–Volterra model using CaliPro. **(A)** Parameter 3–97% percentiles for each iteration with iteration 28 yielding the highest 51% pass rate highlighted in red. **(B)** Trajectories corresponding to the 51% pass rate showing a wide range of matching parameters. Blue curves indicate passing simulations and red curves indicate simulations that failed the CaliPro criteria. **(C)** Parameter space represented as a t-SNE plot showing tight grouping between pass and fail parameter sets. Blue and red points indicate passing and failing simulations, respectively. Although a t-SNE plot is not necessary for only two parameters, we use this representation to analyze higher-dimensional parameter space exploration in the immune-HIV-1/AIDS model. **(D)** Separated density plots of pass and fail parameter sets of **(C)**.

When we use CaliPro for this set of dependent parameters even for this non-complex model, we see a limitation of global LHS sampling: the *α* and *β* parameters are dependent, but LHS assumes the sampled parameters are independent. We show the parameter ranges adjusted by CaliPro oscillate between two very similar ranges (Figures 7, 8). The way to work around this issue of parameter dependence is to simply fix one of the parameters and calibrate the other. Nevertheless, we show this calibration result of varying both parameters to directly compare with the calibration result from the ABC-SMC method.

**FIGURE 9:**
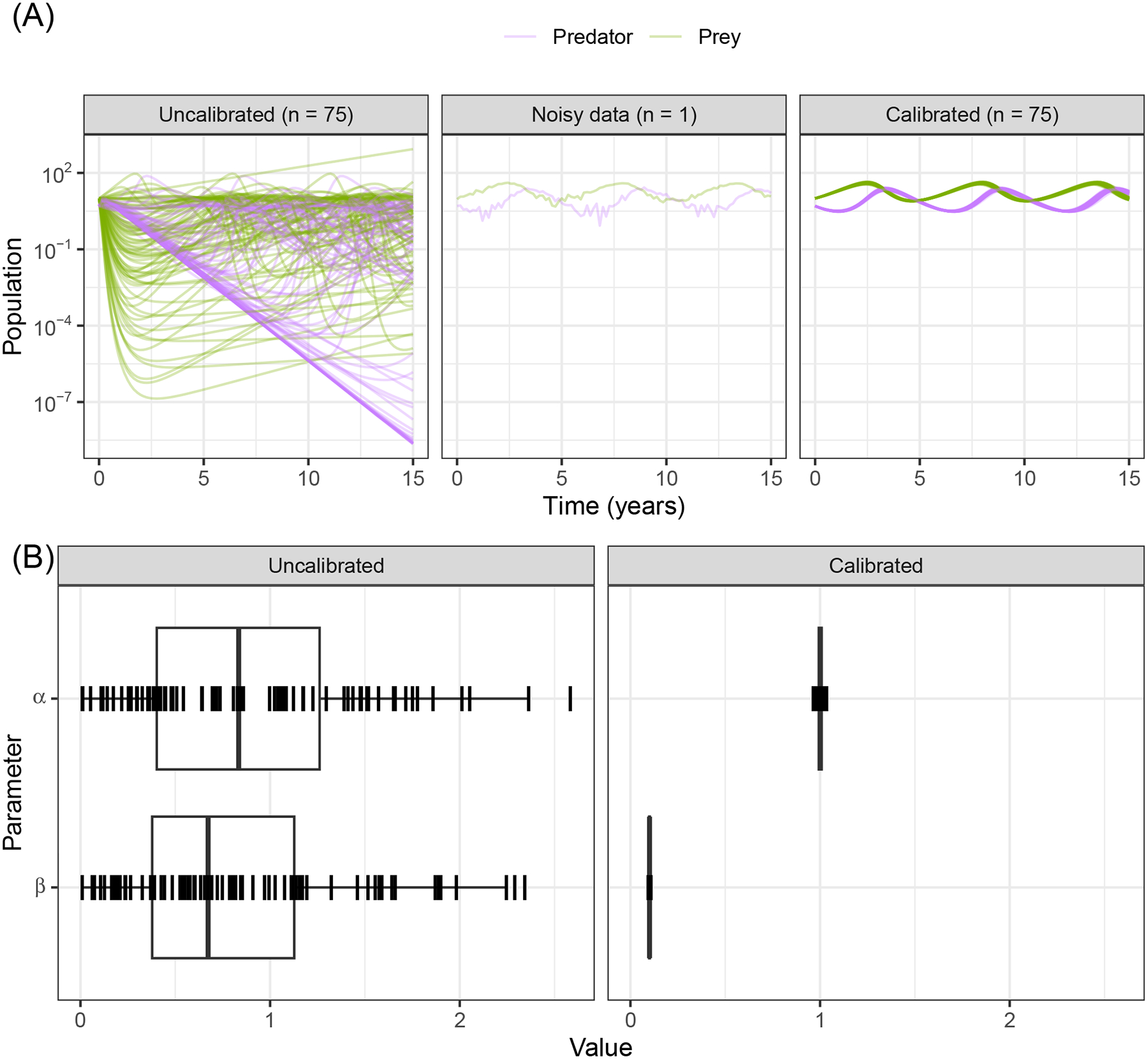
Calibration of the Lotka–Volterra model using ABC-SMC. **(A)** We sample from the prior distributions to simulate model outcomes (before fitting to noisy data), and from the posterior after calibration using ABC-SMC. Purple curves are predator populations and green curves are prey populations. Out of 2,000 samples, 75 translucent curves of predator-prey population counts illustrate calibration to the noisy data points. **(B)** The broad half-Normal priors for the uncalibrated parameters *α* and *β* after calibration are sampled very close to the true dataset values of 1.0 and 0.1, respectively.

For ABC-SMC, we sample 2000 samples for each iteration until SMC beta convergence (Figure 9A). We subsample trajectories from parameters before and after calibration. The calibrated parameters are much closer to the noisy dataset from using the distance function rather than the wider CaliPro boundaries, and the expected value of the *β* parameter is closer to the 0.1 value used to generate the noisy data (Figure 9B).

#### 3.1.2. Immune-HIV-1/AIDS model

The four equation model of immune-HIV-1/AIDS infection [41] offers additional complexity over Lotka–Volterra model as it has 8 parameters and also oscillatory regions of parameter space. The oscillatory regions are challenging for the solver and the solver will often fail for combinations of parameters that produce sharp oscillations. Therefore, a calibration method needs to be resilient to sampled parameter combinations that result in incomplete or unavailable simulations. The model is:

$$\frac{dT}{dt}=s-\mu_{T}+rT\left(1-\frac{T+T_{li}+T_{ui}}{T_{\text{max}}}\right)-k_{1}VT\;\frac{dT_{li}}{dt}=k_{1}VT-\mu_{T}T_{li}-k_{2}T_{li}\;\frac{dT_{ai}}{dt}=k_{2}T_{li}-\mu_{b}T_{ai}\;\frac{dV}{dt}=N\mu_{b}T_{ai}-k_{1}VT-\mu_{V}V$$

where,

T≡ Uninfected *CD*4^+^ cells

$T_{li}\equiv$ Latently infected *CD*4^+^ cells

$T_{ai}\equiv$ Actively infected *CD*4^+^ cells

V≡ HIV cells

s≡ Rate of supply of *CD*4^+^ cells from precursors (day^−1^mm^−3^)

r≡ Rate of growth for the *CD*4^+^cells (day^−1^)

$T_{\text{max}}\equiv$ Maximum *CD*4^+^ cells (mm^−3^)

$\mu_{T}\equiv$ Death rate of uninfected and latently infected *CD*4^+^ cells (day^−1^)

$\mu_{b}\equiv$ Death rate of actively infected *CD*4^+^ cells (day^−1^)

$\mu_{V}\equiv$ Death rate of free virus (day^−1^)

$k_{1}\equiv$ Rate constant for CD^+^ becoming infected (mm^3^ day^−1^)

$k_{2}\equiv$ Rate latently to actively infected conversion (day^−1^)

N≡ Number of free viruses produced by lysing a *CD*4^+^ cell (counts)

Initial values:

$$T\left(0\right)=T_{0}=1000\;T_{li}\left(0\right)=T_{li,0}=0\;T_{ai}\left(0\right)=T_{ai,0}=0\;V\left(0\right)=V_{0}=1000$$

Priors:

s~Gamma(k=1.99,θ=5.68) [day^−1^mm^−3^]

$r\sim \text{Gamma}\left(k=4.53,\theta =6.99\times 10^{-3}\right)$ [day^−1^]

$T_{\text{max}}=1500\left[{\text{ mm}}^{-3}\right]$ (fixed)

$\mu_{T}\sim \text{Gamma}\left(k=2.11,\theta =0.01\right)$ [day^−1^]

$\mu_{b}\sim \text{Gamma}\left(k=1.99,\theta =0.136\right)$ [day^−1^]

$\mu_{V}\sim \text{Gamma}\left(k=1.99,\theta =1.36\right)$ [day^−1^]

$k_{1}\sim \text{Gamma}\left(k=1.98,\theta =1.35\times 10^{-5}\right)$ [day^3^mm^−1^]

$k_{2}\sim \text{Gamma}\left(k=1.59,\theta =0.002\right)$ [day^−1^]

N~ Negative-Binomial (*n* = 13.5, *p* = 0.0148) [counts]

The immune-HIV-1/AIDS model that we originally published used uniform distribution boundaries for all parameters. However, to make the Bayesian and CaliPro approaches comparable, we treated the bounds of the uniform distributions as percentiles to fit the gamma and negative-binomial distributions so that the sampler could more widely explore parameter space. In addition to the oscillatory regions of parameter space, this wider parameter space is an additional challenge imposed rather than the approach of detuning parameters that we used in the previous example.

**FIGURE 10:**
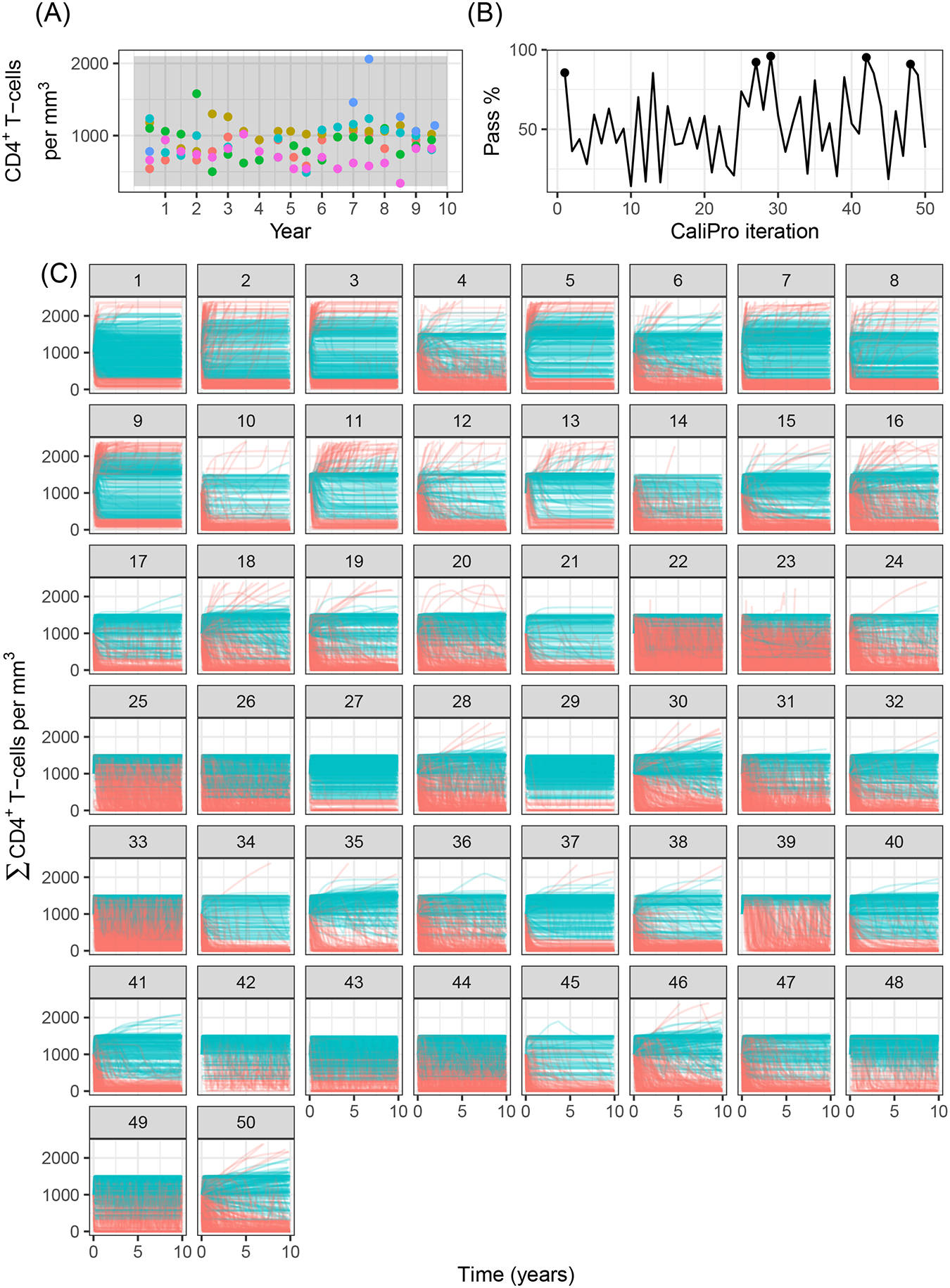
Calibration trajectories of the immune-HIV-1/AIDS model using CaliPro. **(A)** Colored dots represent longitudinal data from 6 patients from which we establish the pass criterion requiring the simulation trajectory to be within the clinical range of 300 to 2100 CD4^+^ T-cell counts shown with the gray shading. **(B)** The simulation pass rate across the 5 replicates starts at 83–92% and therefore the parameters do not require calibration, but we still simulate CaliPro to explore holes in parameter space. The method shows resilience in recovering high calibration values at iterations 27, 29, 42, and 48. **(C)** We color trajectories of simulations blue if they pass (i.e., are within the clinical range of 300 to 2100 CD4^+^ T-cell counts) and red if they fail (i.e., any point in the trajectory is outside the clinical range).

**FIGURE 11:**
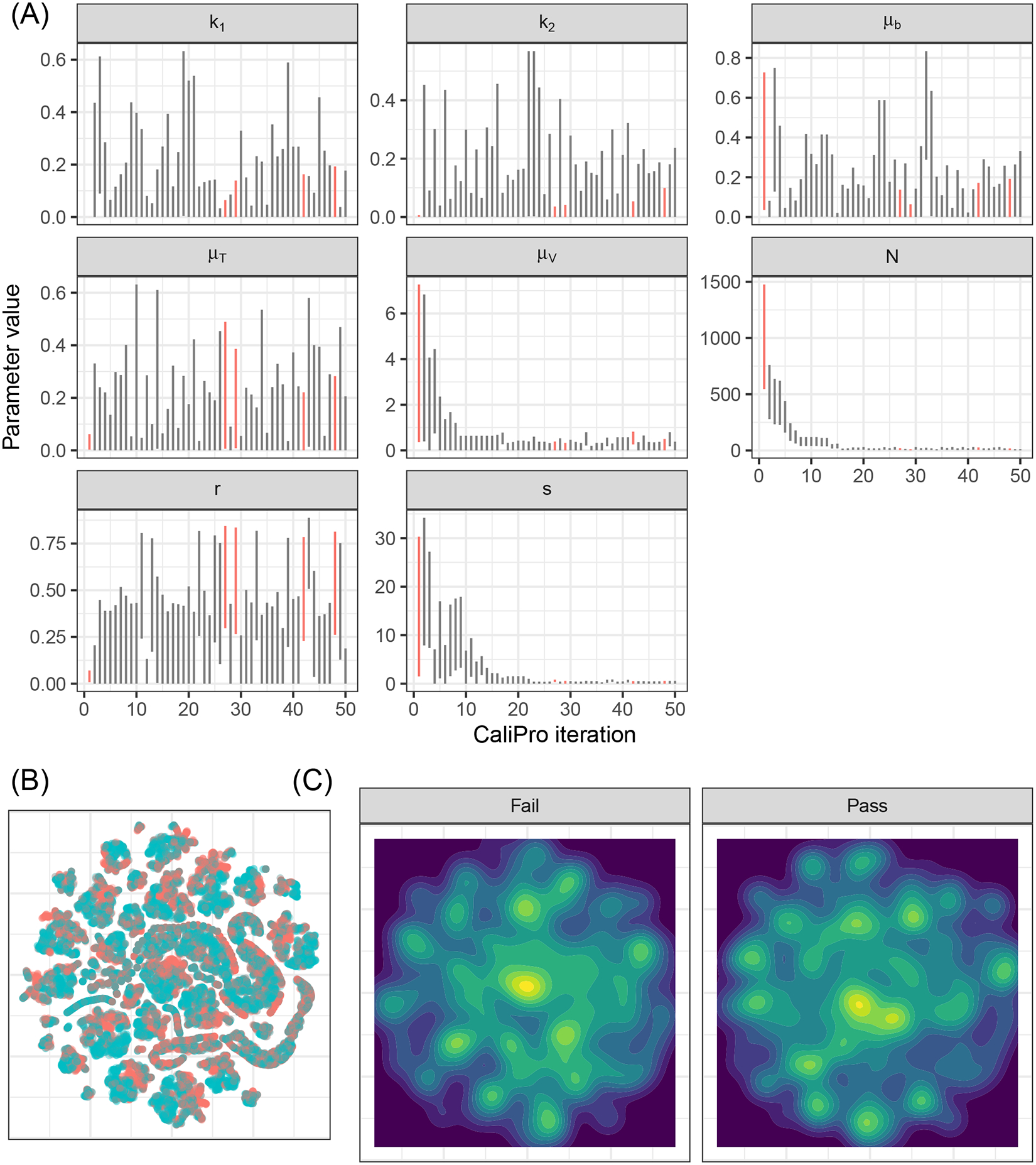
Calibration parameters of the immune-HIV-1/AIDS model using CaliPro. **(A)** Parameter 3–97% percentiles for each iteration. Parameter combinations with more >90% pass percentage are highlighted in red. **(B)** t-SNE plot of all parameters sampled colored by their pass or fail result (blue or red, respectively) showing the complexity of the parameter space. (C) Separated density plots of pass and fail parameter sets.

Applying CaliPro classifies simulations into pass and fail sets to adjust parameter ranges, and these classifications are based on user discretionary boundaries that fit the reference data [14]. We overlay shows the uninfected CD4^+^ T-cell counts of 6 patients [42] with the CaliPro Boolean pass-fail region surrounding all the tracks rounded to the nearest hundred counts, namely 300 and 2100 (Figure 10A). Across the 5 LHS replicates, 83−92% of simulations pass using this criterion without making any adjustments and therefore we do not need to further calibrate as it risks overfitting the model. Nevertheless, to better understand how CaliPro and ABC methods handle the immune-HIV-1/AIDS model with its problematic regions of outcome space, we simulated CaliPro for 50 iterations so that the parameter fitting can be compared against ABC. CaliPro was able to identify parameter ranges with >90% passing simulations at four later iterations (at iterations 27, 29, 42, and 48) indicated by the peak dots in Figure 10B, that summarize the pass-fail graphs in Figure 10C, and the corresponding parameters highlighted in Figure 11A. The grouping of pass and fail simulations in parameter space is shown in Figures 11B, C.

**FIGURE 12:**
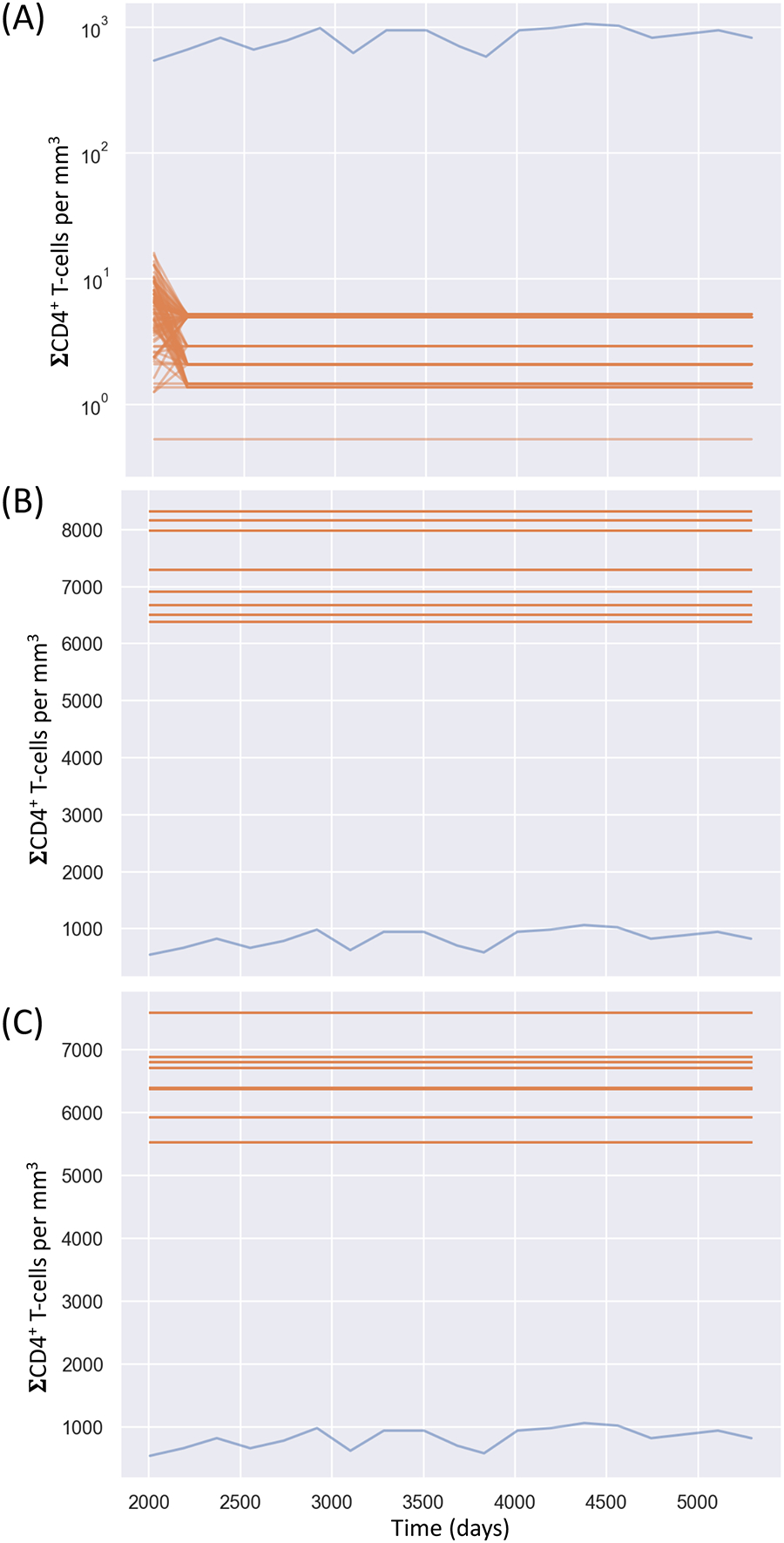
Calibration of the immune-HIV-1/AIDS model using ABC-SMC. (**A**) Comparison of posterior draws when varying all parameters, (**B**) varying four parameters (and fixing $\mu_{⊤}=0.02,r=0.03{\text{ k}}_{1}=2.4\times 10^{-5},{\text{k}}_{2}=3\times 10^{-3}$), and (**C**) varying three parameters (by also fixing $\mu_{\text{b}}=0.24$ ). Patient data is shown with the blue line, and simulated samples after calibration with the orange lines.

Applying ABC, the parameters settled on were nearly a magnitude away on either side of the reference patient data even when fixing most of the parameters to values we know do not produce oscillations (Figure 12). One reason for this may be that the kernel parameter updates are less tolerant than CaliPro to any missing model simulations, caused by failing with certain sets of parameters. To handle missing simulations, the ABC calibration framework may need to assign infinite distances to incomplete simulations and treat those specifically when computing the next proposed parameters in the SMC chains. Together, these two ODE examples shed light on the strengths and weaknesses of these methods when applied to dependent parameters and to models with holes in parameter space. We compare the two approaches in more detail next.

### 3.2. Calibration of stochastic models

Comparing the CaliPro-LHS and ABC-SMC methods on identical complex stochastic models is beyond the scope of this work. Use of these methods separately on stochastic models been previously described as follows. CaliPro-LHS has been used to calibrate the *GranSim* stochastic agent-based model that captures formation of lung structures called granulomas during infection with *Mycobacterium tuberculosis* [14]. ABC-SMC has been used to calibrate a tumor spheroid growth stochastic agent-based model [43] and stochastic models of cell division and differentiation [44].

Stochastic models have both aleatory and epistemic uncertainty. Aleatory arises due to uncertainty in parameter estimates, and additional uncertainty (epistemic) arises from stochastic components of the model. We have talked mostly about aleatory in this work; however, the main difference when calibrating stochastic models, is this epistemic uncertainty. Thus, there is an additional requirement to use the same parameter set but the model must be simulated with different random number generator seeds at least 3–5 times and then model outputs aggregated. This reduces the epistemic uncertainty. To prevent any additional complexity in the calibration code, the model executable itself can wrap the underlying replicates and aggregation so that the calibration code only sees the aggregated model outputs in the same dimensionality as model outputs without using any replicates [see [14] as an example of this].

### 3.3. Comparison of CaliPro with ABC-SMC

We present a summary of the differences between CaliPro and ABC in Table 3. We also point to Figure 2 that further elucidates a decision flowchart for choosing between these methods or choosing them amongst the landscape of other available methods. Table 3 and Figure 2 provide a comprehensive comparison of CaliPro and ABC.

One significant difference between CaliPro and ABC, is the implementation difference of employing global or local sampling to explore parameter space when proposing parameter ranges. CaliPro typically uses Latin Hypercube Sampling (LHS), which is a global sampling technique [45]. ABC starts with global sampling using the initial priors and then progressively updates the priors using local sampling with sequential Monte Carlo weight adjustment; the weight adjustment methods are often called particle filtering or importance sampling. Global sampling using rejection sampling [46] is a simple method used with ABC but is generally considered too slow to be practical. Therefore, the approaches of CaliPro and ABC have complementary strengths: ABC is guaranteed to converge to the true posterior distribution with sufficient samples, but CaliPro requires fewer iterations and employs global sampling for all iterations.

The advantages of using CaliPro include a reduced risk of overfitting to partial experimental data by setting the pass constraints to accept simulated values that fall within ranges of experimental data. Secondly, CaliPro is often used with global parameter sampling such as Latin hypercube sampling (LHS) and therefore samples broadly from parameter space [45], which more robustly captures wider ranges of experimental outcomes. Lastly, CaliPro is resilient to holes in outcome space because such outcomes are classified into the fail set to inform future sampling.

## 4. Discussion

Calibration of complex models often needs to be performed when building complex models or when adding equations or reparametrizing. Both CaliPro and ABC rely on pseudo-likelihood to tune model parameters so that they capture full ranges of biological and clinical outcomes. As we detail in Table 3, CaliPro’s use of binary constraints make it possible to encode any number of constraints from experimental and synthetic datasets. In larger models, applying these constraints is often done in stages: once the pass rate is sufficiently high, more stringent constraints can be applied at later stages of calibration. However, the system of binary constraints also limits CaliPro, but not limit ABC. Using CaliPro, a single “bad” parameter value rejects the entire parameter set whereas the local search of ABC-SMC particle weighting can help adjust and improve the “bad” parameter value. Said another way, the discrete binary encoding of CaliPro is not smooth and can propose narrower parameter sets than ABC. However, the ADS and HDR functions smooth these discrete pass-fail results into adjusted parameter ranges. Lastly, ABC is more resilient to getting stuck on local maxima; CaliPro relies on using replicates to mitigate the effects of local maxima.

The two different examples we discussed highlights cases where one method performs better than the other. CaliPro’s LHS assumes independent parameters. When calibrating the two dependent parameters of the predator-prey model, CaliPro oscillates between two sets of these two parameters highlighting the behavior one may encounter where the parameters being sampled violate the parameter independence assumption. Fixing one of the dependent parameters to calibrate the other is necessary in such a case. Conversely, ABC-SMC assumes complete data and seems to have trouble with not-a-number (NaN) model output values. Some of these errors were mitigated by using the simpler Metropolis–Hastings kernel of PyMC instead of the default multivariate Normal kernel. CaliPro accommodates such incomplete simulation output by assigning such parameters into the fail set for updating parameter ranges for the next iteration. Thus, CaliPro is resilient to such discontinuities in parameter space.

The SMC sampler in ABC makes the technique useful for slow models and/or exploring high dimensional parameter space, because unlike most other Bayesian samplers where adding more chains serve only to check intra- and inter-chain parameter variance, each SMC chain adjusts particle weights to effectively explore more parameter space. Frameworks like pyABC further offer the unique feature of adaptively spawning more chains to minimize sampler wall time.

We encountered several practical challenges with using ABC software. Surprisingly, even when starting from well-behaved, published parameter ranges of the immune-HIV-1/AIDS model, the pyABC software would never complete calibration, even though 75–84% of simulations passed CaliPro’s more relaxed Boolean criterion of data-fitting. We encountered two sources of failures with pyABC: “prior density zero” errors during sampling, and memory resource exhaustion due to limited control of the adaptive population samplers. PyMC, another ABC-SMC framework, was able to complete calibration but we had challenges troubleshooting errors when attempting complex distance functions to multiple patient timeseries trajectories because computation is deferred until much later during execution making it difficult to relate error messages to the relevant code. The complex implementations of both pyABC and PyMC makes it difficult to reason about sources of model fitting errors. Therefore, the immune-HIV-1/AIDS model example highlights the simplicity and usefulness of CaliPro for earlier stages of parameter inference.

Tuning stochastic models requires aggregating additional model simulations to reduce epistemic uncertainty on parameter tuning. As mentioned, the simplest way to integrate stochastic models into calibration frameworks is to make the stochasticity blind to the calibration framework by wrapping the model replicates to produce aggregated output with the same dimensionality as unaggregated output.

Further work for developing CaliPro would entail improving the numerical complexities with fitting small distributions with draws close to zero. To work around the numerical stability of fitting these small draws to distributions, we used rescaling factors, but appropriately using rescaling factors is specific to the type of distribution being fit. Rather than using rescaling factors, an alternative approach that can be used in such probability algorithm implementations is to convert to log-scale for fitting and converting back after fitting. Besides fitting small distribution values, another complexity arising from using the optimizer is choosing useful initial values of the distribution parameters. More work is necessary to automatically choose initial values or find an off-the-shelf software library that provides this feature. Such work toward improving the distribution tuning method of using both percentiles and draws allows CaliPro to meaningfully tune non-uniform parameters from a limited number of simulations.

## Figures and Tables

**TABLE 1:** Keyword descriptions of model and parameter estimation concepts.

Concept grouping	Model and parameter estimation concepts
Model implementations	1. **Multi-scale**: A mixture of models at different spatial resolutions and/or different temporal resolutions. They typically have mixtures of discrete and continuous data, and different implementation approaches making them hybrid models. 2. **Hybrid**: Models containing multiple formulations such as ordinary differential equations, discrete, or stochastic components that must be evaluated concurrently to simulate the system of interest. 3. **Dynamical**: Models with a time dimension. 4. **Mechanistic**: Models where the underlying mechanism is known or can be approximated without entirely relying on probabilistic events. 5. **Probabilistic**: Models that do not require knowledge of mechanisms but instead rely on propagating probability distributions of parameters. Although the models discussed in this paper are mechanistic, calibrating these complex models uses a probabilistic process.
Model parameters	6. **Structural identifiability**: Whether a parameter can be uniquely estimated when fitting the model to experimental datasets [3, 4]. 7. **Parameter calibration**: Tuning parameter boundaries or distributions to capture broad ranges and types of reference experimental datasets. 8. **High-dimensional parameter space**: Large numbers of parameters that characterize a complex model. Also called a multidimensional hypercube. 9. **Summary statistics**: Numerical description of parameter samples or experimental datasets. For example, mean, median, and variance describe a single variable, and correlations and covariance describe a pair of variables. 10. **Sufficient summary statistics**: Sufficient summary statistics contain all available information of the original distribution [5]. Sufficient summary statistics are not necessarily parameters of the distribution (such as the mean, standard deviation, shape, scale, etc.) because they can summarize conditional distributions. More formally, a distribution is sufficient if itcan be Fisher-Neyman factorized. 11. **Dimensional reduction**: Creating summary statistics of high dimensions to aid comparisons between model outcomes and experimental datasets. 12. **Prior distribution**: Fixed model parameter probability distribution from *a priori* knowledge or experimental data that, ideally, was obtained earlier and therefore independent from data generated within a current study. 13. **Posterior distribution**: Probability distribution specified by Bayes theorem combining the prior distributions with a model and experimental datasets.
Parameter sampling	14. **Global search**: Sampling parameter space simultaneously rather than iteratively using previously sampled regions. 15. **Local search**: Sampling parameter space of interest in relation to previous sampled regions. 16. **Particle**: Multiple parameter probability density functions intersecting at a point in parameter space. 17. **Filtering**: Resampling particles to generate parameter distributions. 18. **Markov process**: A data smoothing technique that assigns samples to an underlying state machine. Such a model infers the underlying state, and smoothing is created by a state (probabilistically) remaining unchanged as opposed to transitioning to another state with different data generation characteristics. 19. **Confidence/credible intervals**: Range of parameter values with an associated sampling probability.
Objective functions of sampling parameter space	20. **Likelihood**: The joint probability of the observed data and the model. For example, the likelihood using the model of fair dice yielding an observed roll of 18 dice showing all 5s is ($\frac{1}{6}$)^18^, whereas a model where all the dice have 5s on all sides would have absolute certainty with a likelihood of 1 for the same observation of the dice. 21. **Pseudo-likelihood**: Any objective function that does not require direct numerical evaluation of the likelihood. Approximates the unknown likelihood function with a distance function or kernel density estimator based on simulations of the model. This approach is often chosen when computing the likelihood function is intractable. 22. **Approximate Bayesian computing(ABC)**: Typical Bayesian inference fully evaluates the model likelihood function, or partially evaluates the likelihood function when only comparing its ratio. ABC instead avoids numerically computing the likelihood function by simulating model data and comparing it against experimental data using pseudo-likelihood. 23. **Boolean function**: A function which outputs only true and false values.

Several definitions are summarized for background completeness.

**TABLE 2:** Globally sampling parameters evenly using the cumulative probability density transformation.

LHS sample combination	Parameter 1 (Uniform distr.)	Parameter 2 (Log-unif. distr.)	Parameter 3 (Normal distr.)
1	96	3.12	84
2	72	2.36	99
3	196	2.96	58
4	155	4.89	116
5	12	4.59	131

In Latin hypercube sampling (LHS), combinations of random parameter samples without replacement are used to globally sample model outcomes, such as the five-sample combination shown in Figure 4. Although only five samples are shown to explain the principle of LHS, in practice many more samples than the number of varied parameters would be used to reach high levels of accuracy although there is no agreed upon guideline for choosing the number of samples; see Marino et al. [21] for more details.

**TABLE 3:** Conceptual differences between CaliPro and the probability density approximation variant of approximate Bayesian computing.

Property	CaliPro	ABC
Sampling	Global: typically, Latin hypercube sampling (LHS)	Local: typically, sequential Monte Carlo (SMC)
Pseudo-likelihood	Binary: classification into pass-fail sets	Continuous: distance or kernel function
Filtering	ADS or HDR using pass-fail sets	Weights of pseudo-likelihood particles
Tuning	Coverage threshold (only if using HDR)	Epsilon or kernel distance
Convergence	Absolute pass rate, typically between 75–90%	Relative autocorrelation threshold
User discretion	Constraint choices	Sufficient summary statistics
Use cases	1. Wide range of outputs 2. Avoid summary statistics 3. Intractable regions of outcome space	Avoid local maxima

## Data Availability

The original contributions presented in the study are archived on Zenodo [32]. Further inquiries can be directed to the corresponding author.
